# The Fear of COVID-19: Gender Differences among Italian Health Volunteers

**DOI:** 10.3390/ijerph19116369

**Published:** 2022-05-24

**Authors:** Jessica Burrai, Alessandro Quaglieri, Umberto Aitella, Clarissa Cricenti, Ivan D’Alessio, Alessandra Pizzo, Giulia Lausi, Anna Maria Giannini, Emanuela Mari

**Affiliations:** Department of Psychology, Sapienza University of Rome, 00185 Rome, Italy; jessica.burrai@uniroma1.it (J.B.); alessandro.quaglieri@uniroma1.it (A.Q.); aitella.1712631@studenti.uniroma1.it (U.A.); clarissa.cricenti@uniroma1.it (C.C.); ivan.dalessio@uniroma1.it (I.D.); alessandra.pizzo@uniroma1.it (A.P.); giulia.lausi@uniroma1.it (G.L.); annamaria.giannini@uniroma1.it (A.M.G.)

**Keywords:** anxiety, stress, depression, posttraumatic stress disorder, COVID-19 and mental health, well-being, volunteer personnel, death, coronavirus pandemic

## Abstract

Background: During the COVID-19 pandemic, the fear of being infected was a major concern, resulting in both physical and psychological effects. Despite several studies on fear of COVID-19 in the general population, the effects on healthy volunteers who face COVID-19 on the frontlines have not yet been investigated. Methods: An online survey on specific psychological variables related to COVID-19 was administered to 720 healthy volunteers, and gender differences were investigated. Results: The primary finding was that females showed higher scores in all dimensions assessed. A multiple linear regression conducted on both genders exhibited a similar pattern of predictors, highlighting the pivotal role of negative affect in the male group. Conclusions: The findings suggest that COVID-19 had significant effects on healthy volunteers, especially in the female group. Although the previous literature did not report the crucial role played by the negative affect in the male sample, these results highlight the need to deepen how both genders use different emotional strategies to cope with stressful situations. This study may be useful in the development of specific psychological support and ad hoc training for healthy volunteers.

## 1. Introduction

The COVID-19 pandemic has affected daily life, bringing about negative consequences on a physical and psychological level. Studies [1,2] that investigated the spread of COVID-19 in health care professionals found an increase in the risk of contracting COVID-19 among this population due to work environment and overcrowding in hospitals. Health and frontline workers were the most exposed to the development of the fear of COVID-19 [3,4,5,6,7], even with respect to their own family members contracting the virus [8,9]. Most of the literature [10,11,12,13,14,15] has investigated mental health in health care workers; however, only a few studies [16,17,18] have investigated the effects of the pandemic on the psychological health of humanitarian volunteers who play an important role in the management of emergencies. In fact, during the COVID-19 pandemic, as well as in past epidemics [19], the overload of intensive care units for the management of the critically ill has challenged national health systems. Moreover, the rapid expansion of COVID-19 resulted in the need for a rapid readjustment of health policies in different countries, and this had a great impact in terms of medical liability in respect to the management of “non-COVID” conditions: in fact, the quality of services and the performance of health care personnel was greatly affected by the fear of COVID-19 [20,21], exposing them to a number of allegations of suspected malpractice [22]. Government management agencies in several countries around the world have adopted volunteer systems to provide medical and psychosocial aid to the population of patients and to support the health system: nurses [23], professionals or students of health professions, such as medicine [24] and psychology [25], and citizens [26].

The 2002 United Nations General Assembly defined volunteering as “a wide range of activities, including traditional forms of mutual aid and self-help, formal service delivery and other forms of civic participation, undertaken of free will, for the general public good and where monetary reward is not the principal motivating factor” [27,28]. Volunteering can therefore be expressed as a form of unpaid work that has social and economic value and results from an individual’s free choice to serve [29,30].

Volunteering can take several forms distinguished by five descriptive components: (1) structure, which can be formal, where activities are carried out through specific organisations, or informal, if activities are carried out spontaneously outside registered organisations; (2) site, where activities can be carried out online or offline; (3) intensity, through which activities can be carried out episodically or regularly; (4) aspiration, whether volunteering focused on self-development or community building; and (5) category, whether the activity carried out is “service”, formal activities aiming at responding to the needs of a community; “mutual aid”, informal activities aiming at responding to a shared problem; “participation”, in which volunteers are involved in decision-making mechanisms at different levels; “campaigning”, with the aim of performing prevention and promotion activities; and “leisure”, which is expressed in activities related to personal interests, such as art [28].

In order to address the wide range of needs that have emerged as a result of the current pandemic, several activities aligned with their expertise have been carried out by volunteers: training, prevention, and promotion of preventative behaviours from COVID-19 [31], education on behaviours to be implemented for people who have tested positive for COVID-19, contact tracing [23], telemedicine for patients with COVID-19 for continuity of care [32] and telepsychology to provide psychological first aid and psychoeducation services [25], delivery of essential goods such as food and medicine [26,33] or distribution of face masks [34], and telephone support and online social activities for isolated individuals or patients with COVID-19 [24,26,33].

People engage in volunteer activities primarily to provide aid and contribute to the fight against COVID-19. As reported by a nurse who volunteered in the United States during the first phase of the COVID-19 emergency: “This situation was new for everyone, and the uncertainty of what was happening was scary […] the opportunity to volunteer provided me with a sense of pride in doing good for others and the community to do something to impact the spread of COVID-19” [23].

Despite the limitations imposed by COVID-19, volunteering in Italy has never declined but rather has continued to move forward and “reinvent itself” within the community. It has given its indispensable contributions to the distribution of necessities (food, hygiene products, clothing, medicines), helping those facing economic difficulties, organising fundraisers, listening, and maintaining a relationship with the community and the vulnerable through the use of IT tools. This has led, in many cases, to an increase in the amount of work, as opposed to a decrease. Regardless, volunteers are committed to the training necessary to acquire COVID-19-related health skills [35].

Several studies have been conducted on the general population [36,37,38,39], on the psychiatric population [40,41], and on some categories of workers [42] to investigate a possible increase in the occurrence of anxiety disorders, stress, depression, fear of COVID-19, etc. However, few studies have dealt with volunteers who provided some essential activities and services during the pandemic.

### 1.1. Gender Differences in Psychological Outcomes

Several studies have investigated gender differences in mental health in the general population after the spread of the COVID-19 pandemic. Specifically, the pandemic was found to have a more pronounced effect on women, who reported a higher prevalence and severity of depression, anxiety, and symptoms of posttraumatic stress disorder, such as insomnia [43] and avoidance [44], than men [11,43,44,45,46,47,48,49,50,51]. These findings are supported by a study conducted in 59 countries around the world that also find that women are more likely than men to use a variety of adaptive coping strategies, such as proactively seeking assistance or obtaining emotional support from others [52]. This might explain why females also appear to have experienced greater improvements in mental health over time. Females initially scored significantly higher than males on various measures of mental well-being but later improved, which may imply a fading of gender differences as an indicator of greater emotional distress [43,44]; a lack of adaptive coping strategies in men may mask the presence of greater symptoms of anxiety and difficulty coping in males [43,45]. Finally, women have appeared to report lower expectations of the health consequences of COVID-19 and a greater fear of personal death than men, which may be regarded as a significant predictor of anxiety symptoms [53]. Regarding volunteers specifically, several studies have also shown that women are more likely to participate in volunteer activities than men [34,54,55,56,57,58].

The limited number of studies that have investigated gender differences in mental health among health care workers during the COVID-19 pandemic found, consistent with the data presented above in the general population, that female workers showed a higher prevalence of depression, anxiety, stress, and insomnia symptoms than men [59,60,61]. Specifically, female volunteers appear to exhibit greater physical and emotional stress symptoms, and gender appears to be predictive of arousal, avoidance, and intrusion symptoms of stress [62]. However, women seem to use coping strategies, such as “stopping unpleasant emotions and thoughts”, that may serve to reduce the hyperactivation and intrusiveness aspects of the trauma memory and fear of being infected by the virus and infecting family members. In addition, fear of contagion appears to be related to personality variables, such as neuroticism or emotional instability [50]. Again, it is important to note that other coping strategies have also emerged over time, such as problem-focused coping, used primarily by males, most likely due to a greater understanding of emergency and prevention measures, which seem to have a positive effect on physical stress only [63].

### 1.2. Study Aims and Hypothesis

The aim of the present study is to investigate, by using an online questionnaire, whether perceptions related to the dangerousness of SARS-CoV-2 (e.g., fear of COVID-19) may be associated with a worsening of psychological well-being and whether these effects may be influenced by gender differences in Italian health volunteers.

In particular, we sought to investigate possible gender differences related to fear of COVID-19.

In particular, we expected:

**Hypothesis** **1** **(H1).** *Significant differences between male and female health volunteers on COVID-19 exposure experience, depression, anxiety, stress and coping strategies, fear of COVID-19, fear of death, and posttraumatic stress disorder related to COVID-19*.

**Hypothesis** **2** **(H2).** *Significant correlations between fear of COVID-19 and the investigated variables depending on gender.*

**Hypothesis** **3** **(H3).** *Different variables will be predictors of fear of COVID-19 in the male and female groups.*

## 2. Materials and Methods

### 2.1. Participants

A total sample of 720 participants (Table 1) was involved in the study, and 50% were males (i.e., a total of 360) with an age range from 18 to 72 years (M = 48.83; SD = 12.63). A total of 49.9% of the sample was from northern Italy, 48.5% from central Italy and 1.7% from southern Italy. With respect to marital status, 26.5% were single, 57.1% were married or in a civil union, 14.7% were separated or divorced, and 1.7% were widowed. With respect to educational level, 11% had a middle school degree, 52.2% had a high school degree, 11.5% had a Bachelor’s degree, 16.5% had a Master’s degree, and 8.8% had a Postgraduate degree. The majority of respondents lived with their families (76.5%), while fewer participants lived on their own (19.6%) or had a roommate (3.9%).

Regarding the COVID-19 experience, 17.8% of respondents experienced COVID-19 symptoms, while 82.2% did not experience symptoms. With respect to the item “Who contracted COVID-19?”, 54.7% stated “him/herself”, 30.5% reported relatives or friends, and 14.8% declared cohabitants had contracted it. With reference to vaccination, the majority of respondents reported that they received at least one dose (95.8%). All respondents were members of voluntary associations, such as the Italian Red Cross, the Civil Protection, and the National Confederation of the Misericordia of Italy.

### 2.2. Procedure

Data were collected from 2 September 2021 to 20 December 2021 (in Italy, this period represented the turn of the third and fourth COVID waves; in particular, the fourth COVID wave had brought the largest infections among the Italian and international population, registering weekly peaks of more than 1700 cases per 100,000 inhabitants). Participants completed a secure online survey optimised for its use on computers, tablets, and mobile devices. The Qualtrics Survey Platform was used to distribute the questionnaire widely throughout Italy. A non-probabilistic and convenience sampling technique was used in order to successfully attract as many voluntary participants as possible, motivated by interest and curiosity about the research topic.

The questionnaire was distributed through different channels, such as authors’ official working platforms, by word of mouth, and through social networks. After providing informed consent, each respondent was able to voluntarily decide to join the research and start answering the digital survey. Participants were informed that data collection would be performed anonymously and would not be shared outside the research procedure. This study was conducted in accordance with the ethical standards of the Helsinki Declaration and was approved by the Institutional Review Board of the Department of Psychology of “Sapienza” University of Rome protocol number 1444/2021.

### 2.3. Materials

For the present study, an online questionnaire consisting of different sections was developed. First, there was a short summary of demographic data (e.g., age, gender, living conditions); then, the questionnaire included the following measures.

#### 2.3.1. COVID-19 Exposure Experience

This scale was developed by the authors to investigate the individual experience with respect to the spread of the virus and the restrictive containment measures through items such as “In your professional or volunteering framework, how much have the interpersonal relationships with colleagues changed since the pandemic started?” or “How much attention do you pay to any COVID-19-related symptoms (e.g., cough, difficulty breathing, fever)?”. The 10 items were evaluated on a 5-point Likert scale ranging from 1 (“Strongly disagree”) to 5 (“Strongly agree”).

#### 2.3.2. Depression Anxiety Stress Scales (DASS-21)

The DASS-21 is a set of three self-report scales widely used to measure emotional states of depression, anxiety, and stress [64]. Each of the three DASS-21 scales includes 7 items: the Depression scale assesses dysphoria, hopelessness, devaluation of life, self-deprecation, lack of interest/involvement, anhedonia, and inertia; the Anxiety scale relates to autonomic system arousal, skeletal muscle effects, situational anxiety, and subjective experience of anxious affects; the Stress scale assesses the presence of nonspecific levels of arousal, difficulty relaxing, nervous excitement, irritability, agitation, hyperactivity, and impatience. The rating scale is as follows: 1 (“Did not apply to me at all”), 2 (“Applied to me to some degree, or some of the time”), 3 (“Applied to me to a considerable degree or a good part of time”), 4 (“Applied to me very much or most of the time”). The Italian validation of the DASS-21 [65] was used. In this study, Cronbach’s alpha was 0.89, 0.79, and 0.89 for depression, anxiety, and stress, respectively.

#### 2.3.3. The Fear of COVID-19 Scale

The questionnaire consists of 10 items investigating the fear triggered by the possibility of developing the disease caused by the SARS-CoV-2 virus [66]. Responses were evaluated on a 5-point Likert scale ranging from 1 (“Not at all”) to 5 (“Extremely”). The higher the score, the greater the fear of COVID-19. The Italian validation of the Fear of COVID-19 Scale [67] was used. In this study, Cronbach’s alpha was 0.85.

#### 2.3.4. Post-Traumatic Stress Disorder Related to COVID-19 Questionnaire

The PTSD Related to COVID-19 Questionnaire is a self-report validated measure designed to specifically assess symptoms regarding the risk of PTSD in the current pandemic emergency [68]. The questionnaire assesses seven dimensions: Intrusion, Avoidance, Negative Affect, Anhedonia, Dysphoric arousal, Anxious arousal, and Externalising behaviour. All 19 items refer to the seven days prior to completion during the SARS-CoV-2 outbreak. Responses were evaluated on a 5-point Likert scale ranging from 0 (“Not at all”) to 4 (“Extremely”). With respect to the dimensions with a two-item factor structure, we adopted Spearman–Brown reliability, and the results showed coefficients of 0.7, 0.59, and 0.60 for avoidance, dysphoric arousal, and anxious arousal, respectively. Other dimensions showed Cronbach’s alpha values of 0.78, 0.68, 0.74, and 0.84 for intrusion, negative affect, anhedonia, and externalising behaviour, respectively. Cronbach’s alpha for the total score was 0.92.

#### 2.3.5. The Collett-Lester Fear of Death Scale

The scale provides a measure of death anxiety, distinguishing between the fear of death and the fear of dying, each referring to oneself and the others [69]. Four dimensions are assessed: your own death, your own dying, the death of others, and the dying of others. It consists of 28 items, and responses are evaluated on a 5-point Likert scale ranging from 1 (“Not at all”) to 5 (“Very much”). Higher scores indicate a greater fear. The revised version [70], adapted with translation and back translation into the Italian language, was used. In this study, Cronbach’s alpha was 0.87, 0.88, 0.83, and 0.87 for Own Death, Own Dying, Others Death, and Others Dying, respectively.

#### 2.3.6. Brief COPE Inventory

The Brief COPE Inventory assesses different coping strategies or cognitive adjustments in response to stressful events [71]. The scale measures individuals’ coping styles through the identification of three dimensions of coping: problem-focused strategies, emotion-focused coping, and dysfunctional strategies. The scale is composed of 28 items with scores measured on a 4-point Likert scale ranging from 1 (“Almost never”) to 4 (“Almost always”). The Italian validation of the Brief COPE Inventory [72] was used. In this study, Cronbach’s alpha was 0.81, 0.63, and 0.60 for problem-focused strategies, emotion-focused coping, and dysfunctional strategies, respectively.

### 2.4. Data Analysis

Statistical analyses were performed using Statistical Package for Social Science (SPSS; version 26.0; IBM SPSS, Armonk, NY, USA). Descriptive statistics are provided for gender, age, education, marital status, profession, family type, and COVID-19-related items. Questionnaire scores were analysed by using an independent-samples *t* test. When Levene’s test for equality of variances was significant, we reported the corrected degree of freedom rounded to the nearest whole number (as implemented in SPSS). For each gender separately, we calculated Pearson correlations and performed linear regression analyses to study the relationships between variables and fear of COVID-19. The variance inflation factor (VIF) and tolerance were used to verify the problem of multicollinearity.

The sample size was determined through an a priori power analysis, performed using G*Power3 to test differences between two independent means, with an alpha of 0.05 to achieve a power of 0.95. The effect size (d = 0.80 “large effect” as implemented in G*Power3) was a priori defined. The result yielded a total sample size of 84 participants. 

Statistical significance was defined as *p* < 0.05. The distributions of all data were verified for normality, and all statistical analyses were performed on de-identified data.

## 3. Results

**Figure 1:**
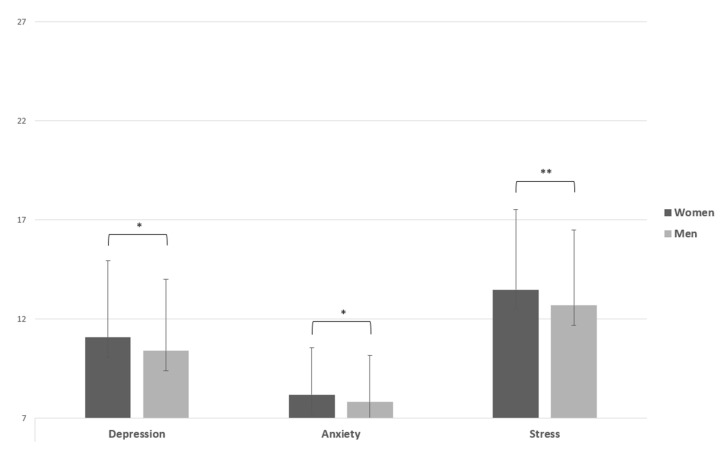
Independent sample *t* test of DASS-21. Note: * = *p* < 0.05; ** = *p* < 0.01.

With respect to the “Depression” dimension of DASS-21, an independent *t* test indicated that scores were significantly higher for women (M = 11.07, SD = 3.87) than for men (M = 10.39, SD = 3.60), t(718) = −2.45, *p* < 0.05, d = 0.18. The “Anxiety” dimension of DASS-21 showed a statistically significant result t(718) = −2.10, *p* < 0.05, d = 0.15, in which women reported a higher mean score (M = 8.19, SD = 2.36) than men (M = 7.82, SD = 2.35). The score on the “Stress” dimension was higher for women (M = 13.48, SD = 4.04) than men (M = 12.69, SD = 3.79), t(718) = −2.70, *p* < 0.01, d = 0.20 (Figure 1).

**Figure 2:**
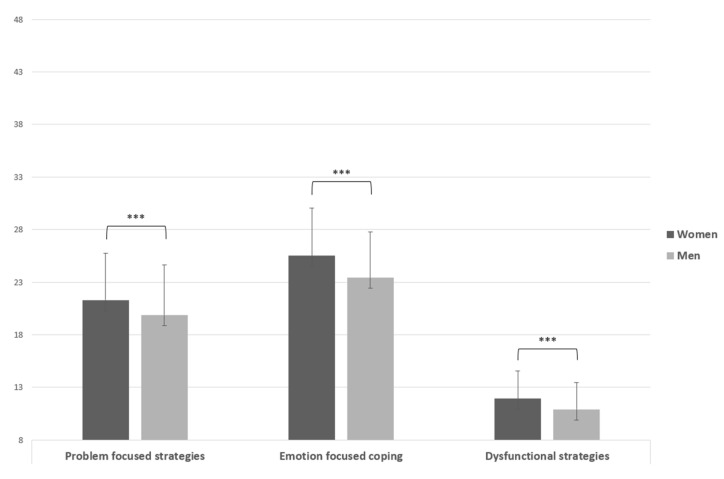
Independent sample *t* test of Brief-coping Inventory. Note: *** = *p* < 0.001.

The Brief-coping Inventory showed statistically significant results in all three dimensions considered; the score of “Problem focused strategies” was higher for women (M = 21.30, SD = 4.42) than for men (M = 19.85, SD = 4.77), t(718) = −4.21, *p* < 0.001, d = 0.31, and “Emotion focused coping” showed a higher mean for women (M = 25.50, SD = 4.52) than for men (M = 23.43, SD = 4.37), t(718) = −6.22, *p* < 0.001, d = 0.46. Last, the “Dysfunctional strategies” dimension reported a higher mean score in women (M = 11.94, SD = 2.61) than in men (M = 10.91, SD = 2.54), t(718) = −5.32, *p* < 0.001, d = 0.39 (Figure 2).

**Figure 3:**
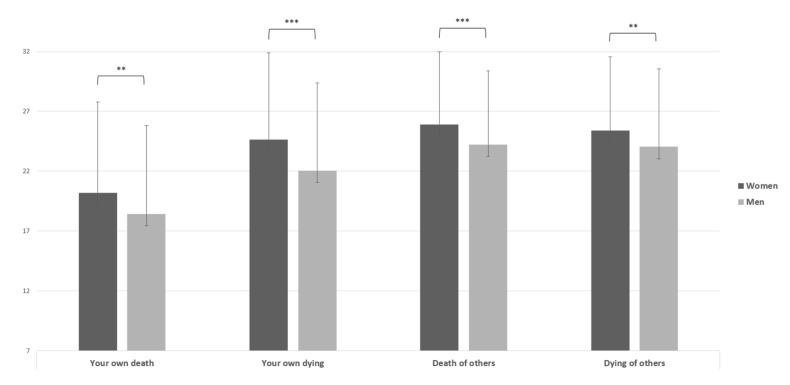
Independent sample *t* test of Collett-Lester Scale. Note: ** = *p* < 0.01; *** = *p* < 0.001.

With respect to all dimensions of the Collett-Lester scale, the results showed higher mean scores in the female group (M = 20.18; 24.65; 25.91; 25.38) and the male group (M = 18.42; 22.05; 24.23, 24.03), t(718) = −3.15, *p* < 0.01, d = 0.23; t(718) = −4.78, *p* < 0.001, d = 0.035; t(718) = −3.69, *p* < 0.001, d = 0.027; t(718) = −2.84, *p* < 0.01, d = 0.021 for “Your own death”, “Your own dying”, the “Death of others”, and the “Dying of others”, respectively; Figure 3).

**Figure 4:**
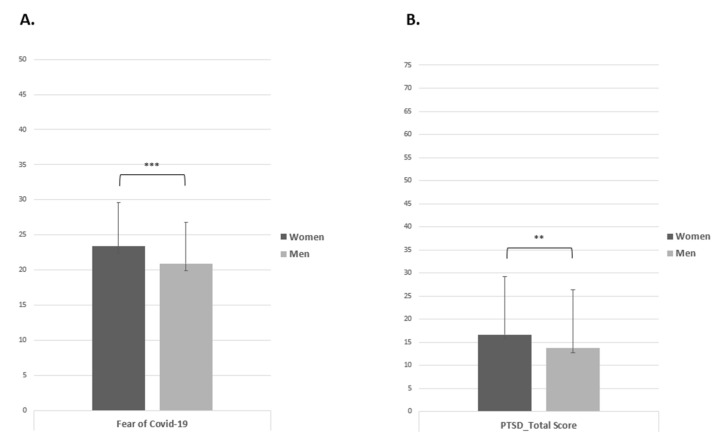
Independent sample *t* test of Fear of COVID-19 Scale (**A**) and PTSD total score. (**B**). Note: ** = *p* < 0.01; *** = *p* < 0.001.

Concerning the “Fear of COVID-19” scale, there was a statistically significant result in which the female group reported a higher mean (M = 23.39, SD = 6.25) than the male group (M = 20.90, SD = 5.84), t(718) = −5.51, *p* < 0.001, d = 0.41 (Figure 4).

**Figure 5:**
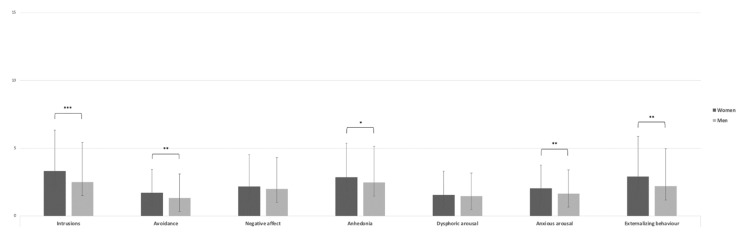
Independent sample *t* test of PTSD Related to COVID-19 subscales. Note: * = *p* < 0.05; ** = *p* < 0.01; *** = *p* < 0.001.

The PTSD total scale showed a statistically significant result in women, with a higher mean score (M = 16.68, SD = 12.48) than in men (M = 13.72, SD = 12.61), t(718) = −3.17, *p* < 0.01 d = 0.023 (Figure 4). With respect of the PTSD Related to COVID-19 Questionnaire, the women group showed higher scores in the “Intrusions” (M = 3.33, SD = 3.03), t(718) = −3.69, *p* < 0.001, d = 0.027), “Avoidance” (M = 1.73, SD = 1.73), t(718) = −2.94, *p* < 0.01, d = 0.021), “Anhedonia” (M = 2.88, SD = 2.50), t(718) = 2.03, *p* < 0.05, d = 0.014), “Anxious arousal” (M = 4.00, SD = 1.71), t(718) = −2.93, *p* < 0.01, d = 0.021), and “Externalising behaviour” (M = 2.92, SD = 2.98), t(718) = −3.32, *p* < 0.01, d = 0.025) dimensions than the men group (M = 2.50, SD = 2.92; M = 1.35, SD = 1.78; M = 2.49, SD = 2.68; M = 1.67, SD = 1.76; M = 2.21, SD = 2.77, respectively). The “Negative Affect”, and “Dysphoric arousal” dimensions showed no statistically significant results (*p* = 0.278, d = 0.08; *p* = 0.505, d = 0.04; Figure 5).

Pearson’s correlation analysis showed that the fear of COVID-19 score was significantly and positively correlated with all variables for each gender separately. To clarify, in the female group, the fear of COVID-19 score showed a moderate positive correlation with the intrusions, avoidance, negative affect, anhedonia, and anxious arousal dimension of the PTSD scale and a low positive correlation with other variables considered. With respect to the male group, fear of COVID-19 showed a moderate correlation with the intrusions, avoidance, negative affect, and anxious arousal dimension of the PTSD scale and a low positive correlation with the other variables considered.

In addition, the significant correlation coefficients of the two groups were compared with Fisher’s *z* test. For clarity, we report all correlations and statistical results concerning both the male and female groups in Table 2.

Subsequently, we tested the prediction concerning the link between fear of COVID-19 by using a stepwise linear regression. We conducted a separate linear regression for each gender; the results of the regression concerning the male group indicated that the four predictors explained 31.0% of the dependant variable’s variance (R^2^ = 0.31, F _(4,355)_ = 40.44, *p* < 0.001). It was found that Negative Affect significantly predicted the Fear of COVID-19 (β = 0.67, *p* < 0.001), as did “Own Death” (β = 0.15, *p* < 0.001), “Intrusions” (β = 0.29, *p* < 0.05), and “Anxious Arousal” (β = 0.41, *p* < 0.05; Table 3).

Concerning the regression of the female group results, the five predictors explained 41.0% of the dependant variable’s variance (R^2^ = 0.41, F _(5,354)_ = 49.25, *p* < 0.001). It was found that “Anxious Arousal” significantly predicted the Fear of COVID-19 (β = 1.12, *p* < 0.001), as did “Dying Other” (β = 0.17, *p* < 0.001), “Intrusions” (β = 0.38, *p* < 0.01), “Own Death” (β = 0.09, *p* < 0.05), and last, “Negative Affect” (β = 0.29, *p* < 0.05; Table 4).

## 4. Discussion

Health volunteers played a key role in managing the pandemic emergency by being an important resource, not only in Italy but also in other countries (e.g., Thailand, Australia, and India; [57,73,74]). To our knowledge, no study has investigated the same variables considered in the same reference group, except for a few (e.g., the association between volunteerism, mental distress, and happiness in a comparison between Chinese and American volunteers and health professionals [75]).

The present study examined the differences between men and women in a sample of Italian health volunteers regarding the psychological impact of COVID-19. In particular, the research underlined the specific characteristics of the two groups in the possible association between fear of COVID-19 and other psychological features.

In general, it has been shown that increased fear of COVID-19 is associated with some psychological variables.

Our first hypothesis (H1) was confirmed. In fact, the results underlined significant differences between males and females: female volunteers showed higher means in all dimensions of the DASS (Stress, Anxiety, and Depression). These data are concordant with the scientific literature present for both the general population [13,76,77,78,79,80] and the specific volunteer population [75], in which women show higher levels of depression, anxiety, and somatisation symptoms; however, these differences are small, probably because of the period of data collection (the second pandemic wave). In fact, some studies found that gender differences in psychological well-being faded as time went by [43,44]; we can imagine that these differences were slowly fading, and future studies may investigate if these differences will be still present at the end of the pandemic situation.

Compared to coping strategies, female volunteers use more problem-focused and emotion-focused coping strategies than male volunteers; at the same time, however, they also show higher scores for the use of dysfunctional strategies, such as denial and minimisation of the stressful event with a medium effect size on these differences. On this point, the scientific literature shows that while positive coping strategies, such as problem-solving focused strategies, would act as a mediating factor on the incidence of stress and burnout in COVID-19 volunteers and in particular, it would seem to act as a protective factor against the development of post-traumatic stress [63,81,82], volunteers who used dysfunctional coping strategies have high levels of stress, burnout, and depressive symptoms [82,83].

Female volunteers also show higher scores than men in fear of COVID-19, with a medium effect size; indeed, a systematic review and meta-analysis [84] conducted on 44 articles with a sample size of 52,462 shows that the fear of COVID-19 is higher in women than in men. This finding may also be explained by the fact that it is more acceptable for women to express their fears of illness [85]. Furthermore, a low Fear of COVID score could be closely related to the decision to continue the activities of volunteers.

Another variable we considered in our study is fear of death; for this variable, female volunteers also show higher scores in all dimensions of the scale, which is consistent with a large body of research on both female adolescents and adults [86,87]; also, in this case, the effect size is medium (above 0.2). The research of Silva and colleagues [88] shows that levels of fear of death as a psychological disposition are a strong predictor of lower well-being and that this relationship is mediated by anxiety regarding COVID-19; furthermore, the most innovative aspect of their research is the evidence that the intensity of worry individuals have with the imminence of death and the negative relationship they have with the idea of their own death. This is relevant to understanding the psychological states of increased anxiety and sadness during a pandemic. Previous studies have also suggested that mortality salience would be a particularly important predictor of these psychological variables [89].

Furthermore, female volunteers showed higher means in some dimensions of PTSD related to COVID-19 (“intrusions”, “avoidance”, “anhedonia”, “anxious arousal”, and “externalising behaviour”) with differences in their effect sizes. Particularly, except for anhedonia, whose effect size is small, all the other dimensions showed medium effects. These findings are coherent not only with the trauma literature, which suggests that the risk of PTSD is twice as high among women as among men [90], but also, more recently, research suggests that women may be at a higher risk of developing PTSD following infectious diseases than men. Indeed, the recent COVID-19 literature has highlighted gender differences in PTSD among health care workers [91] and in the general adult population [13,79,92]. However, it is important to note that both groups show moderate values but not above the cut-off of 26 [68], which may not rule out a possible development of PTSD; it has been seen as in Portugal et al. [93] that the level of self-perceived stress due to isolation from family members for a health care worker is a significant and important factor in the severity of PTSD and depressive symptoms during the COVID-19 pandemic. Indeed, in future studies, it would be appropriate to investigate the construct with additional tools (e.g., clinical interview).

Our second hypothesis (H2) was also confirmed. Fear of COVID-19 correlated with all investigated variables except Problem-focused strategies of the Brief COPE Inventory.

In particular, for the Male Group, the analysis underlined positive correlations between Fear of COVID-19 and Depression Anxiety Stress Scales (Anxiety, Depression, and Stress), Collett-Lester Fear of Death Scale (Own Death, Own Dying, Death Others, Dying Others), Brief COPE Inventory (Emotion-focused coping and Dysfunctional strategies), and Post-Traumatic Stress Disorder Related to COVID-19 Questionnaire (Intrusions, Avoidance, Negative Affect, Anhedonia, Dysphoric Arousal, Anxious Arousal, Externalising Behaviours).

Similarly, in the female group, fear of COVID-19 was positively correlated with the same variables as for the male group. Between the two groups, statistically significant differences in correlations were found only on the Anhedonia and Anxious Arousal PTSD subscales; the significant difference between the two groups in the correlations (regard to the PTSD subdimensions) could suggest that anhedonia and anxiety are factors characterising fear of COVID-19 in women. These data were also highlighted in Simsir et al. [94], a meta-analysis of 33 studies, which showed that fear of COVID-19 was strongly correlated with anxiety, traumatic stress, and distress, as well as moderately correlated with stress and depression.

Our third hypothesis (H3) about different variables predicting fear of COVID-19 in the two groups was partially confirmed. In fact, the first predictor of females is Anxious arousal of the PTSD scale, which seems to play a significant role in exacerbating the Fear of COVID-19; this dimension is also found in the male group, but with a significantly lower incidence. This finding seems to be consistent with the literature on the issue, which shows that women have higher levels of anxiety regarding the COVID-19 pandemic [43,59,60,61]. On the other hand, for men, the factor that most explains the investigated dependant variable (i.e., fear of COVID-19) is negative effect as measured by the PTSD scale; this finding is in contrast to the previous study by Fenollar-Cortés et al. [44], in which it emerges that women have significantly higher scores than men with respect to the variable negative affect. It is important to note that this study refers to the period of isolation from COVID-19 (lockdown), whereas our study is temporarily related to cohabitation with the COVID-19 virus, so it is possible that this variable plays a crucial role in the fear of COVID-19. Furthermore, it could be hypothesised that a sense of resignation is predominant in men, in contrast to women, in whom a greater component of anxiety prevails, which can also be explained by the hypothesis that women seem to be more concerned about the consequences of their health if they become infected. In fact, women experience a greater fear of personal death than men, which would seem to be a significant predictor of anxiety symptoms [53]. In our study, the latter finding would seem to be in contrast, as the variable “Own death” measured with the Collett-Lester Fear of Death Scale appears to be predictive of fear of COVID-19 in both groups; in particular, fear of own death, in the men’s group, seems to have a more relevant place in exacerbating fear of COVID-19. It can be assumed that the assessment conducted in our study, in relation to fear of death, is more detailed than in previous papers [53] since a specific scale was used to assess it (i.e., the Collett-Lester Fear of Death Scale), emphasising the different aspects related to fear of death instead of considering them as a single dimension. On this point, it is important to highlight that one of the variables that characterise only the female group is “Dying Others”. It could be hypothesised that the difference between the two groups may be related to greater anxiety in women and lower coping skills compared to men, both related to their own death and the death of others. Future studies could focus on the different subdimensions of fear of death, focusing on one’s own and others’ death while also keeping in check the variable presence/absence of children, which could play a role in explaining the figure.

In conclusion, in both groups, the intrusion PTSD subdimension seemed predictive of fear of COVID-19. This result is in line with the study by Fenollar-Cortés and colleagues [44], which shows no statistically significant differences between genders for intrusive symptoms assessed by the Impact of Event Scale (IES).

Overall, although men are more likely to experience negative health consequences from COVID-19, women report greater fear and more negative expectations about the health consequences of COVID-19 than men. Women also reported more negative emotional experiences in general during the pandemic, although women had implemented more restrictive protective measures [95].

## 5. Limitations

Some limitations of the present study must be considered. First, the present study only used self-report measures for the assessment of the different investigated variables. In addition, the study sample may have been influenced by selection bias, as the questionnaires were more accessible to certain groups of volunteers; in addition, the online administration of the questionnaire compared with the paper-and-pencil version allows for a wider distribution throughout the territory, but less control over the filling in. Although the dissemination of the questionnaire took place at the central level of voluntary associations, the greatest response occurred in northern and central Italy; this may have influenced the results obtained. The lower volunteer response obtained in Southern Italy also depends on the low number of Italian Red Cross Local Committees in the South compared to the entire national panorama, which created a discrepancy in data collection. On the other hand, regarding the Civil Protection Department, the questionnaire link was sent throughout the country, taking care that it was viewed and disseminated.

As also pointed out by Roncone et al. [83], there can be several explanations for the low adherence to completing the questionnaires on the part of volunteers: 1. the difficulty for volunteers to show themselves to be psychologically “fragile” with the fear of compromising their self-image; 2. the lack of time to complete the questionnaires; and 3. the overall duration of the online assessment.

Other studies [96] on emergency workers have confirmed the professional culture of volunteers who may generally be reluctant to recognise the symptoms of psychological distress, with the fear that it will be interpreted as a sign of weakness and inadequacy with respect to their work.

Moreover, it is necessary to point out that the highest number of COVID cases was found in central and northern Italy.

The research also took place at the turn of the third and fourth COVID waves in Italy, but a longitudinal study would have been better for a stronger dataset.

## 6. Conclusions

The results of the present study contribute new insights to the literature by highlighting significant differences between male and female Italian volunteers in the experience of exposure to COVID-19 by focusing on some psychological variables. Even though the Volunteer category was extensively involved at different stages and in different roles in the management of the pandemic situation, few studies have focused specifically on this sample and investigated whether there were differences between the two genders. A fact not to be underestimated is that the data comparing the two genders show statistically significant results of a worse condition for women than for men volunteers, but this does not imply that men are “better off”. In fact, noting the power of the statistical effect, gender, mainly within psychological well-being, may not be the main factor underlying these differences.

Future studies could be conducted confronting volunteers and other worker categories (e.g., law enforcement officers or health workers) who often perform the same tasks under the same conditions but respond to different contingencies: volunteers choose to do it, and law enforcement officers or health workers are obliged to do it. Finally, there are practical implications: the volunteers would have had and still need targeted psychological support to be able to cope with the situation they experienced in the best possible way, and in addition, it would be very important for them to be trained on certain aspects related, for example, to coping strategies or psychological symptoms to be considered.

## Figures and Tables

**Table 1:** Descriptive statistics of the sample.

Gender	Male	360 (50%)
	Female	360 (50%)
Geographic Area	North-Italy	359 (49.9%)
	Centre-Italy	349 (48.5%)
	South-Italy	12 (1.7%)
Marital Status	Single	191 (26.5%)
	Married/Civil Union	411 (57.1%)
	Legally Separated/Divorced	106 (14.7%)
	Widowed	12 (1.7%)
Education	Middle school	79 (11.0%)
	High school	376 (52.2%)
	Bachelor’s degree	83 (11.5%)
	Master’s degree	119 (16.5%)
	Postgraduate degree	63 (8.8%)
Cohabitation	Living alone	141 (19.6%)
	Living with the family	551 (76.5%)
	Living with roommates	28 (3.9%)
Children	Yes	412 (57.2%)
	No	308 (42.8%)
COVID-19 Experience	Yes	128 (17.8%)
	No	592 (82.2%)
Vaccination	Yes	690 (95.8%)
	No	30 (4.2%)
Total		720 (100%)

**Table 2:** Significant correlations between fear of COVID-19 and the other variables in the male and female groups.

	DASS Anx	DASS Dep	DASS Str	Own Death	Own Dying	Death Others	Dying Others	Prob Str	Emo Cop	Dys Str	Intrusions	Avoid	Neg aff	Anhedonia	Dys Arou	Anx Arou	Exte Beh
Male Group																	
Fear COVID-19	0.328 **	0.266 **	0.256 **	0.372 **	0.317 **	0.348 **	0.298 **	0.077	0.183 **	0.287 **	0.431 **	0.413 **	0.491 **	0.284 **	0.336 **	0.416 **	0.313 **
N	360	360	360	360	360	360	360	360	360	360	360	360	360	360	360	360	360
Female Group																	
Fear COVID-19	0.373 **	0.320 **	0.321 **	0.354 **	.0352 **	0.365 **	0.366 **	−0.033	0.138 **	0.292 *	0.488 **	0.453 **	0.445 **	0.404 **	0.345 **	0.544 **	0.355 **
N	360	360	360	360	360	360	360	360	360	360	360	360	360	360	360	360	360
Comparing Correlations																	
z	−0.686	−0.789	−0.948	0.277	−0.527	−0.26	−1.02	-	0.617	−0.073	−0.96	−0.658	0.787	−1.88	−0.136	−2.231	−0.632
*p*	0.246	0.215	0.172	0.391	0.299	0.397	0.153	-	0.269	0.471	0.167	0.255	0.216	0.034 *	0.446	0.013 *	0.264

Note: M = Male group; F = Female group; DASS Anx = Anxiety dimension of DASS-21; DASS-Dep = Depression dimension of DASS-21; DASS Str = Stress dimension of DASS-21; Prob Str = Problem focused strategies; Emo Cop = Emotion focused coping; Dys Str = Dysfunctional strategies; Avoid = Avoidance; Neg aff = Negative Affect; Dys Arou = Dysphoric Arousal; Anx Arou = Anxious Arousal; Exte Beh = Externalising Behaviours; ** = *p* < 0.01; * = *p* < 0.05

**Table 3:** Stepwise linear regression of fear of COVID-19 predictors in the male group.

	b	SE B	β	*p*
(Constant)	15.41	0.695		<0.001
Negative Affect	0.669	0.152	0.265	<0.001
Own death	0.149	0.038	0.188	<0.001
Intrusions	0.288	0.115	0.144	0.013
Anxious arousal	0.406	0.191	0.122	0.035

**Table 4:** Stepwise linear regression of fear of COVID-19 predictors in the female group.

	b	SE B	β	*p*
(Constant)	13.51	1.07		<0.001
Anxious arousal	1.120	0.188	0.311	<0.001
Dying others	0.167	0.046	0.169	<0.001
Intrusions	0.375	0.110	0.183	<0.01
Own death	0.094	0.038	0.117	0.014
Negative Affect	0.287	0.139	0.108	0.041

## Data Availability

The data presented in this study are available on request from the corresponding author. The data are not publicly available due to privacy considerations.
